# *Bacillus cereus* (EG-Q3) in the Gut of *Ectropis grisescens* Contributes to Host Response to Starvation Conditions

**DOI:** 10.3389/fmicb.2022.785415

**Published:** 2022-04-11

**Authors:** Xiayu Li, Yong Zhang, Linlin Zhou, Tian Gao, Yu Zhao, Song Liu, Qingqing Su, Chaoling Wei, Yunqiu Yang, Yanhua Long

**Affiliations:** ^1^School of Life Sciences, Anhui Agricultural University, Hefei, China; ^2^State Key Laboratory of Tea Plant Biology and Utilization, Anhui Agricultural University, Hefei, China

**Keywords:** gut bacteria, starvation, lipid metabolism, physiological adaptation, mechanism

## Abstract

The gut bacteria of insects play an important role in their nutrition, maintenance, and ecological adaption. *Ectropis grisescens* is the most important leaf-feeding pest in tea gardens in China. In order to explore whether *E. grisescens* adaptation under starvation stress is related to its gut bacteria, we used a culture-independent method to compare the composition and diversity of their gut bacteria under starvation treatment. The results revealed no significant changes in core gut bacteria composition and diversity within 24 h of starvation. However, non-core gut bacterial *Bacillus* increased significantly under starvation conditions. *B. cereus* strain EG-Q3 isolated from the gut of *E. grisescens* in carbon source-selected medium showed the ability to degrade fat bodies from *E. grisescens in vitro* and *in vivo*. Moreover, the fat-lowering ratio of *E. grisescens* fed with *B. cereus* strain EG-Q3 (6.76 ± 1.281%) was significantly higher than that of the control group (3.96 ± 0.801%, *t* = 4.15, df = 8, *p* < 0.01) after starvation for 4 h. These findings suggest that non-core gut bacterial *B. cereus* strain EG-Q3 contributes to host adaptation to starvation. Together, this research provides evidence that *E. grisescens* may benefit from non-core gut bacteria under starvation conditions.

## Introduction

Insects are consumers, and cannot produce the organic matter necessary to satisfy their metabolic needs. Due to seasonal and climate change, food distribution is inconsistent throughout a year, and most insects will be subject to hunger stress during some stage of their lives (Rotkopf et al., 2013). Hunger stress has adverse effects including decreased metabolism (Hietakangas and Cohen, 2009) and reproductive ability (Billings et al., 2018), and may even result in death (Yang et al., 2016). Therefore, insects can enter an anti-stress state to adapt to those adverse conditions. For example, when larvae of *Drosophila melanogaster* (Diptera: Drosophilidae) and *Harmonia axyridis* (Coleoptera: Coccinellidae) are nutritionally challenged, they exhibit cannibalistic behavior (Ahmad et al., 2015). Larvae may enter the pupal stage earlier and may remain in this stage longer with increasing days of starvation (Ballard et al., 2008). Insufficient food also limits insect reproduction (Zhang et al., 2015; Ojima et al., 2018) because reducing reproductive investment under starvation conditions can increase somatic cell maintenance (Elkin and Reid, 2005; García-Roger et al., 2006; Billings et al., 2018). Notably, the studies cited above focused on insect adaptation to hunger stress from only the perspective of their behavior. Other studies have shown that insects secrete Adipokinetic hormone to degrade fat and increase energy metabolism in poor nutritional states (Parkash et al., 2012; Rovenko et al., 2015; Kim and Rulifson, 2004; Lee and Park, 2004).

The gut microbiota is associated with many essential host physiological functions (Sommer and Bäckhed, 2013; Xu et al., 2019; Grenier and Leulier, 2020). With the advent of sequencing technology, many bacteria impacting insect life activities have been found in the guts of a variety of insects (Dillon et al., 2008; Hu et al., 2013; Fang et al., 2013; Ma et al., 2021). Some studies have focused on how insects adapt to different types of stress through gut microbes. For example, *D. melanogaster* reared at high temperatures became more cold-tolerant after receiving transplants of gut bacteria from flies which were reared at low temperatures (Moghadam et al., 2017). *Bemisia tabaci* that contained scattered Rickettsia phenotype showed significantly higher thermotolerance (Brumin et al., 2011). In addition, *Bactrocera dorsalis* inoculated with isolated *Citrobacter* sp. showed greater resistance to trichlorphon, confirming the key role of *Citrobacter* sp. in insecticide resistance (Brumin et al., 2011; Cheng et al., 2017). These illustrate the roles of gut microbes in host adaptation to different types of stress, however, whether gut bacteria contribute to host responses to starvation stress remains unknown.

The tea Geometrid moth *Ectropis grisescens* (Warren) (Lepidoptera: Geometridae) is one of the most destructive tea plant pests throughout the **world** (Zhang, 2001; Antony et al., 2011). These chewing defoliators have both high fecundity and gluttony, and typically cause significant losses to tea crops in terms of both yield and quality (Antony et al., 2011; Nakai and Lacey, 2017). *E. grisescens* often cope with food shortages, therefore, exploring how *E. grisescens* adapt to starvation stress using gut bacteria may provide novel insight. Here, we compared the diversity and abundance of gut bacteria in *E. grisescens* during different periods of starvation and identified a bacterial strain (EG-Q3) with the ability to degrade fat. In addition, we tested the fat body degradation ability of this strain *in vitro* and *in vivo*. Our results will expand our understanding of the physiological roles of gut microbiota.

## Materials and Methods

### Insect Rearing

*Ectropis grisescens* eggs were obtained from stock cultures from the State Key Laboratory of Tea Plant Biology and Utilization, Anhui Agricultural University, Hefei, China (31.86°^°^N, 117.27°^°^E). The collected larvae were reared on tea leaves in transparent boxes in a controlled climate room (22°C ± 1°C; relative humidity 75 ± 10%; 16 h light:8 h dark photoperiod). The tea leaves were cut from branches of tea plants using scissors and inserted into floral foam for storage (Zhang et al., 2021).

### Insect Dissection and DNA Extraction

Larvae were reared on tea leaves until they reached the fifth instar. Then, 250 healthy 5th-instar larvae were selected for starvation treatment for 0, 4, 8, 12, or 24 h.

Ten 5th-instar larvae were pooled to into biological replicates and five biological replicates were established per group. Larvae were surface-sterilized by dipping in 75% ethanol for 15 s and then rinsing twice with sterile water for 15 s each time. Dissecting scissors were used to cut laterally behind the head capsule, and the gut was removed from the cuticle with larval forceps. The whole gut, including contents, was collected and placed in a 2.0 ml microcentrifuge tube for DNA extraction.

Total genomic DNA was extracted from samples using a QIAamp DNA Stool Mini Kit (Qiagen, Hilden, Germany) (Mirsepasi et al., 2014). The quality of the DNA was assessed using electrophoresis in 1.2% (w/v) agarose gel prior to amplification and sequencing.

### Amplification and Sequencing of the V3-V4 Region of the 16S rRNA Gene

Gut bacteria were analyzed by sequencing the V3-V4 region of the 16S ribosomal RNA gene (16S rRNA) using the Illumina NovaSeq platform (Illumina, San Diego, CA, United States). Genomic DNA samples were subjected to PCR for amplification of the V3-V4 regions of the 16S rRNA using the universal primers 338F (5′-ACTCCTACGGGAGGCAGCAG-3′) and 806R (5′-GGACTACHVGGGTWTCTAAT-3′). All PCR reactions consisted of 15 μl of Phusion R High-Fidelity PCR Master Mix (New England Biolabs, Beverly, MA, United States), 2 μM forward and reverse primers, and 10 ng of template DNA. The thermal cycling conditions were as follows: 98°C for 1 min followed by 30 cycles at 98°C for 10 s, 50°C for 30 s, 72°C for 30 s, and 72°C for 5 min. The mixture of PCR products was then purified with a Gel Extraction Kit (Qiagen). After PCR amplification, the samples were sequenced on the Illumina NovaSeq platform and 250-bp paired-end reads were generated. All sequences are available as SRA files at the National Center for Biotechnology Information Sequence Read Archive database (NCBI-SRA) under bioProject PRJNA720281 (SRA accession numbers: SAMN18644841).

### Bioinformatics and Statistical Analysis

The sequences were analyzed using the QIIME software package and were used to compare the relative abundances of bacterial taxa (Caporaso et al., 2010b). Operational taxonomic units (OTUs) were assigned with a 97% similarity cutoff using UCLUST version 1.2.22 (Edgar, 2010). The representative sequence (the sequence with the highest relative abundance) for each OTU was used to build the overall OTU table. The taxonomic classifications of each microbial OTU were assigned using Ribosomal Database Project classifier PyNast and SILVA and UNITE as bacterial 16S rRNA databases (Caporaso et al., 2010). Abundances of OTUs were normalized using a standard value for the sample with the fewest sequences. Subsequent analyses of alpha diversity were performed using this normalized output.

The alpha- and beta-diversity indices were calculated using QIIME V1.7.0. The alpha diversity of the gut bacteria complexes was calculated as two diversity indices (Shannon-Wiener and Simpson’s) and two richness estimators (ACE and Chao 1 index). Rarefaction curves were used to verify the quality and depth of sampling. Principal coordinate analysis (PCoA) with weighted and unweighted UniFrac distance metrics was used to detect differences among microbial community structures (Lozupone and Knight, 2005).

### Enrichment, Screening, and Purification of Lipase Producing Bacteria

Fifty 4th-instar larvae were collected and divided into five groups. Each group was starved for 0, 4, 8, 12, or 24 h, and then immediately soaked in 70% ethanol for 3 min to remove surface bacteria. The dissected guts of the larvae were divided among five grinders with 1 ml of sterile water each. The homogenate was added to 100 ml enrichment medium (Yeast extract 0.20 g/L, NaCl 0.50 g/L, Na_2_HPO_4_ 3.50 g/L, KH_2_PO_4_ 1.50 g/L, MgSO_4_⋅7H_2_O 0.50 g/L, Olive oil 10 ml/L) and then incubated at 200 rpm overnight at 37°C. Twenty-four hours later, 1 ml of the enriched liquid was incubated in new enrichment medium for a second round of enrichment. This step was repeated twice. Finally, 100 μl of enriched liquid was coated on a Rhodamine B tablet (Jette and Ziomek, 1994) and cultured at 30°C for 3 days. The grown colonies were purified three times. The transparent circles from cultured strains were observed by irradiating plates with UV light at 350 nm (Kouker and Jaeger, 1987), and the diameter of each fluorescence circle and colony was measured using Vernier calipers. Strains with certain fat degradation ability were selected based on their H/C value, which represent the ratio of hydrolysis circle diameter (H) to colony diameter (C).

### Identification of Bacterial Isolates

Lipase producing bacteria isolated from *E. grisescens* were identified using 16S rDNA gene sequencing and a series of physiological and biochemical tests. A phylogenetic tree of those 16S rDNA sequences was constructed using MAGA 7.0.

### Validation of Liposomal Degradation by EG-Q3 *in vitro*

The isolated strain, EG-Q3, was incubated in LB medium overnight at 200 rpm and 37°C. The bacterial suspension was then centrifuged to remove the LB medium. The pellet of bacteria cells was then washed with ddH_2_O to further remove residual medium. The bacterial cells were diluted with ddH_2_O to a concentration of OD_600_ = 1.0. Ten test tubes containing 5 ml MSM medium (NaCl 1.00 g/L, (NH_4_)_2_SO_4_ 1.00 g/L, K_2_HPO_4_ 1.50 g/L, KH_2_PO_4_ 0.50 g/L, MgSO_4_⋅7H_2_O 0.50 g/L, pH 7.0–7.5) were divided into three experimental groups (i, ii, and iii) and a blank control. Each experiment was replicated three times. Fat bodies dissected from five *E. grisescens* larvae were mashed in a beaker containing 20 ml ddH_2_O. Then, 1 ml fat body homogenate was aspirated into MSM mediums of groups i and iii using pipettes. The above EG-Q3 suspension (OD_600_ = 1.0) was inoculated into sterilized MSM mediums of groups ii and iii at 4% inoculum volume. All of the test tubes were incubated for 3 days at 200 rpm and 37°C.

### Validation of Liposomal Degradation by EG-Q3 *in vivo*

Bacterial suspension of EG-Q3 (OD_600_ = 1.0) was applied to the surface of tea leaves evenly with a brush and allowed to air dry. Some tea leaves were treated at this step using sterile water instead of bacteria suspension. This treatment was repeated three times. One hundred and thirty-eight 2nd-instar larvae with similar body weight (mean ± SD; 11.021 ± 0.512 mg) were divided into control (I) and experimental (II) groups, and were placed in disposable Petri dishes with tea leaves and fed for 3 days. The tea leaves were renewed daily. In order to observe the colonization of bacteria, nine larvae were randomly selected from each group and their guts were diluted and applied to MYP medium (the appropriate medium for *B. cereus*) (Peng et al., 2001). Then, the remaining 120 larvae were subjected to starvation stress. Each treatment contained five biological replicates with ten larvae per replicate. The body mass of larvae from the two groups was measured after 4 and 12 h starvation using an electric microbalance.

## Results

### Composition and Diversity of Gut Bacteria in Larvae of *Ectropis grisescens* After Starvation

**FIGURE 1:**
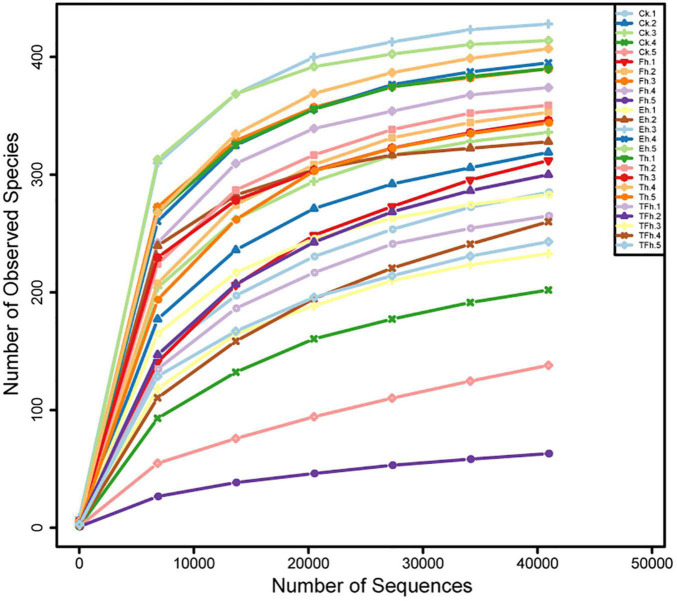
Rarefaction curves based on the numbers of operational taxonomic units (OTUs) observed in the gut microbial communities of *E. grisescens* larvae after 0 h (CK), 4 h (Fh), 8 h (Eh), 12 h (Th), and 24 h (TFh) starvation treatment.

The Illumina NovaSeq sequencing of the bacterial 16S rRNA amplicons from *E. grisescens* with after 0, 4, 8, 12, and 24 h starvation yielded 2,244,492 raw reads in total. After quality filtering and read merging, a total of 1,853,092 high-quality sequences remined. Rarefaction curves clearly demonstrated that the sampling efforts were adequate to fully represent the richness of the gut microbial communities (Figure 1).

**FIGURE 2:**
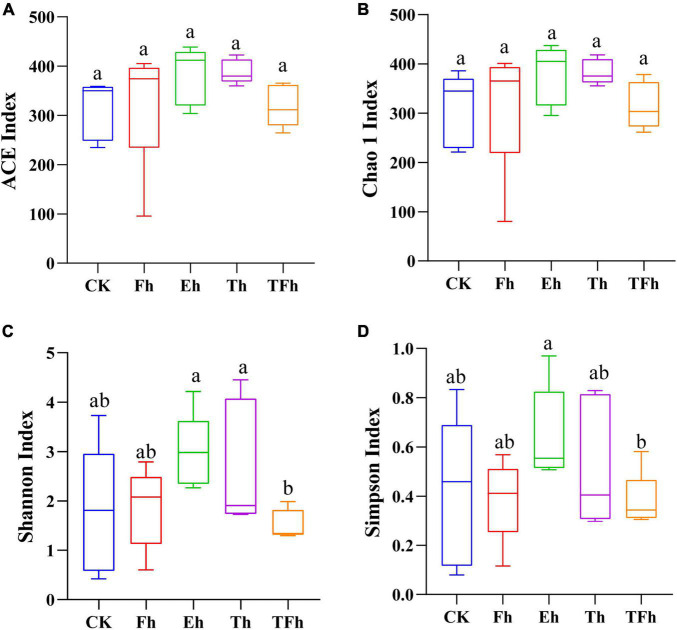
The alpha-diversity based on ACE **(A)**, Chao 1 **(B)**, Shannon **(C)**, and Simpson **(D)** indices of the gut microbial community of *E. grisescens*. Significant differences were detected using unpaired two-tailed *t*-tests. CK: no starvation treatment; Fh: 4 h starvation treatment; Eh: 8 h starvation treatment; Th: 12 h starvation treatment; TFh: 24 h starvation treatment.

The OTU richness (ACE and Chao 1 index) of the gut bacteria did not differ significantly among groups (Figures 2A,B). However, the OTU diversity (Shannon index) of the gut bacteria of larvae exposed to starvation for 24 h (TFh; 1.52 ± 0.27) differed from that of the gut bacteria of larvae exposed to starvation for 8 h (Eh; 2.98 ± 0.68; *t* = 3.99; df = 8; *p* < 0.001) and 12 h (Th; 2.71 ± 1.14; *t* = 2.02; df = 8; *p* < 0.5). In addition, the OTU diversity (Simpson index) of the gut bacteria of TFh (0.38 ± 0.10) differed from that of Eh (0.65 ± 0.17; *t* = 2.66; df = 8; *p* < 0.5) (Figures 2C,D).

**FIGURE 3:**
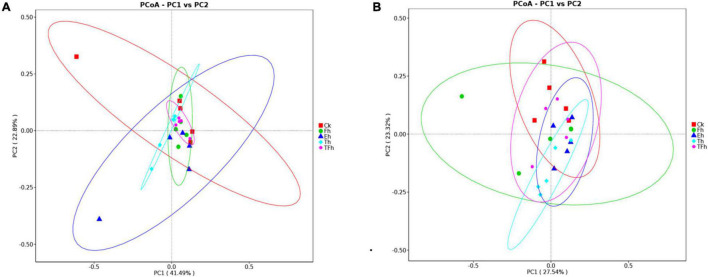
Principal coordinate analysis (PCoA) of bacteria using distances based on weighted **(A)** and unweighted **(B)** UniFrac values.

The PCoA analysis using distances based on weighted and unweighted UniFrac showed that the gut bacterial communities of *E. grisescens* larvae were not distinctive (Figures 3A,B).

**FIGURE 4:**
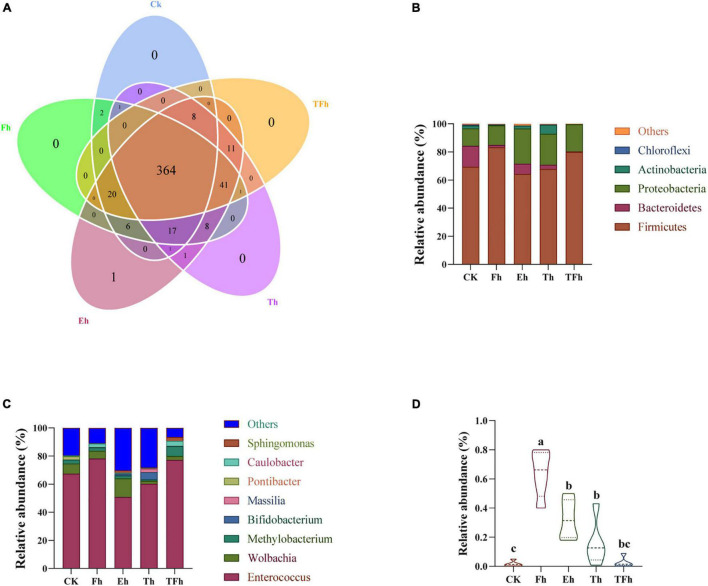
Venn diagram **(A)** of the operational taxonomic units (OTUs) in CK, Fh, Eh, Th, and TFh. Relative abundances are shown for bacteria at the phylum **(B)** and genus **(C)** levels. CK: no starvation treatment; Fh: 4 h starvation treatment; Eh: 8 h starvation treatment; Th: 12 h starvation treatment; TFh: Twenty-four hours starvation treatment. **(D)** The relative abundance of *Bacillus*.

Three hundred sixty-four OTUs were shared by all groups, and accounted for 86.87, 81.79, 79.30, 80.35, and 81.80% of the total OTUs in CK, Fh, Eh, Th, and TFh, respectively (Figure 4A). The dominant bacterial phyla in each group were Firmicutes (50.89–78.12%), Proteobacteria (12.27–25.01%), and Bacteroidetes (0.38–14.95%) (Figure 4B). The dominant bacterial genera in each group were Enterococcus (64.19–79.90%) and Wolbachia (2.13–13.11%) (Figure 4C). The relative abundance of *Bacillus* in CK (0.01 ± 0.17%) differed significantly from that in Fh (0.64 ± 0.14%; *t* = 8.51; df = 8; *p* < 0.0001), Eh (0.32 ± 0.12%; *t* = 5.06; df = 8; *p* < 0.001), and Th (0.16 ± 0.14%; *t* = 2.01; df = 8; *p* < 0.05) (Figure 4D).

### Screening and Identification of the Lipase Producing Strain

**FIGURE 5:**
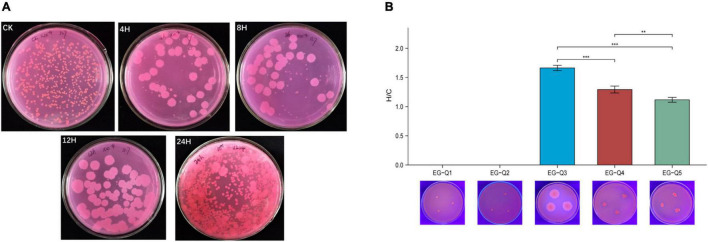
**(A)** The growth of intestinal commensal bacteria on Rhodamine B medium, **(B)** The H/C values and hydrolysis circles of five screened strains.

The gut contents of larvae subjected to starvation for different amounts of time were cultivated on Rhodamine B selective medium, and five potential lipase-producing strains were identified based on colony characteristics (Figure 5A). These strains were named EG-Q1, EG-Q2, EG-Q3, EG-Q4, and EG-Q5.

Isolates of the five strains were inoculated on selective medium again in order to compare their abilities to degrade lipids. Although EG-Q1 and EG-Q2 could be cultured on the selective medium, obvious hydrolysis circles were not observed. The H/C value of EG-Q3 (1.66 ± 0.05) was significantly higher than that of the other strains (Figure 5B). Therefore, EG-Q3 was selected for further *in vivo* and *in vitro* tests.

**FIGURE 6:**
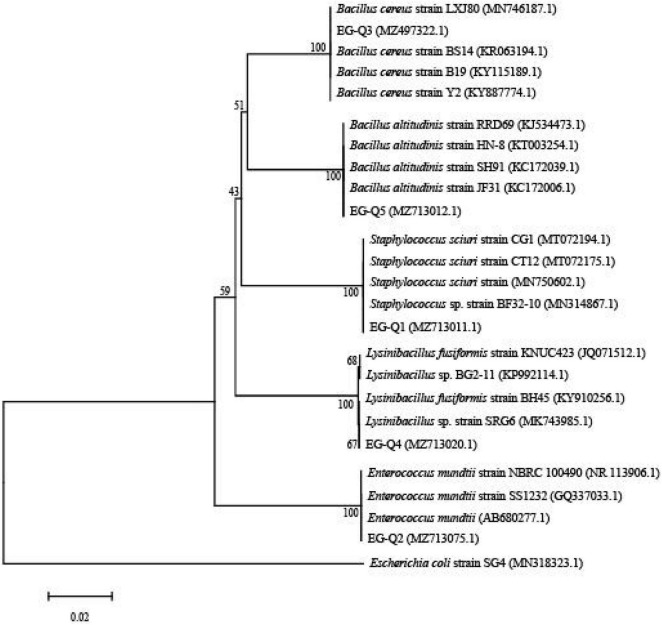
Phylogenetic tree of the five screened strains.

The three isolates producing transparent circles (EG-Q3, EG-Q4, and EG-Q5) were rod shaped Gram-positive bacteria (Supplementary Figure). Through combination with the phylogenetic tree constructed from 16S rDNA sequences of the five strains (Figure 6), EG-Q3 was identified as *B. cereus.* The genome sequence of the isolated strain was registered in GenBank (the Accession Number: MZ497322.1).

### Degradation of Fat Bodies by EG-Q3 *in vitro*

**FIGURE 7:**
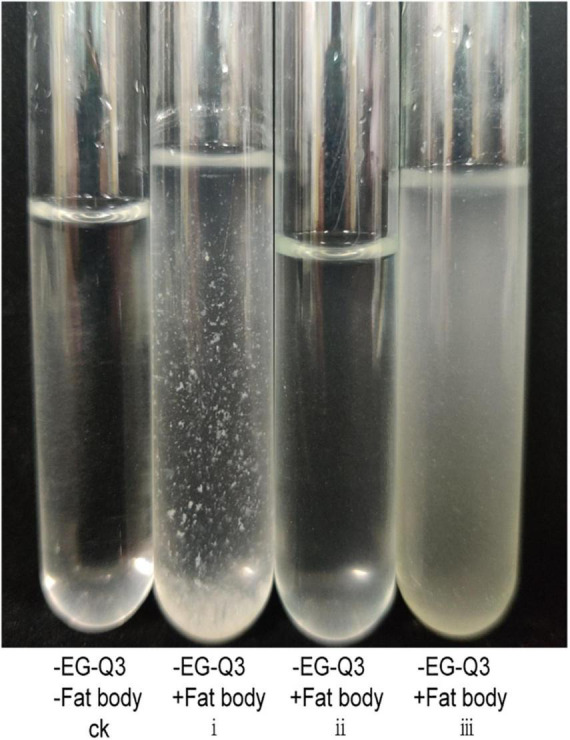
Degradation of fat bodies by EG-Q3 *in vitro*.

In *in vitro* tests of fat body degradation (Figure 7), we found that fat bodies floating in suspension were quite obvious and unbroken in group i. In group ii, although EG-Q3 was inoculated into the MSM medium, the turbidity of the medium did not change, and resembled group CK. However, the fat bodies in group iii were broken and had nearly disappeared, and the MSM medium became significantly turbid. This indicated that EG-Q3 can use fat bodies for proliferation.

### Degradation of Fat Bodies by EG-Q3 *in vivo*

**FIGURE 8:**
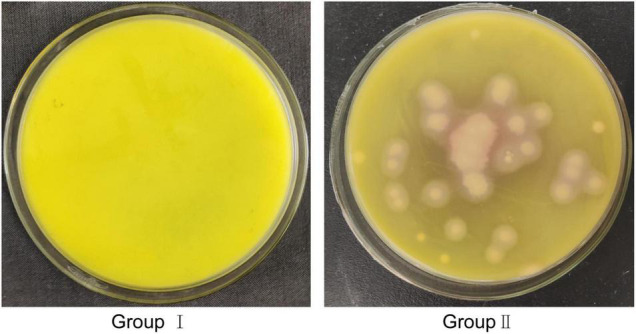
Cultivation of EG-Q3 in *E. grisescens* in MYP medium.

**FIGURE 9:**
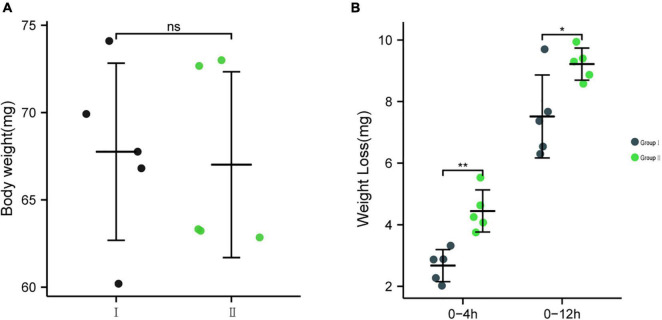
**(A)** The body weights of the two groups of insects before starvation, **(B)** The effect of feeding EG-Q3 on body mass. Independent sample *t*-tests were used to evaluate significance. Data are presented as mean ± SD (*n* = 5). * indicates significance difference between two groups: *, *p* < 0.05; **, *p* < 0.01.

To further determine whether bacteria EG-Q3 could proliferate in the larval gut, tea leaves coated with EG-Q3 were fed to *E. grisescens*. After 3 days of supplementation, EG-Q3 could be detected in the guts of larvae (Figure 8). There was no difference in the body weights of the two groups of insects before starvation (Figure 9A). After confirming that EG-Q3 can colonize larval guts, the larvae were starved and weighed regularly. We found that in group I, the body mass of larvae fed with EG-Q3 decreased significantly after 4 h of starvation (from 67.014 ± 5.318 mg to 62.514 ± 5.519 mg), and the fat-lowering ratio after 4 h of starvation was 6.76%, nearly twice that of group II (3.96%) (Figure 9B). With the extension of starvation time, the weight of the larvae further decreased. The fat-lowering ratio increased to 13.85% after 12 h starvation (control group 11.05%).

## Discussion

Gut bacteria are of increasing interest to entomologists due to their role in mediating insects’ fitness. In this study, a culture-independent approach was used to compare the composition and diversity of gut bacteria under starvation treatment. The results revealed no significant change in gut bacteria composition and diversity within 24 h of starvation. Simultaneously, the composition of core gut bacteria of *E. grisescens* observed in this study was consistent with previous reports (Zhang et al., 2019, 2021). However, the abundance of a non-core bacterial *Bacillus* changed significantly. Therefore, the role of *Bacillus* in the starvation of *E. grisescens* attracted our attention. In this study, we reported the first isolation of *B. cereus* (EG-Q3) associated with host lipid metabolism from the gut of the tea pest *E. grisescens.*

As a multifunctional biocatalyst, lipase has excellent application value and prospects in industries such as biodiesel, food and beverage, leather, textile, detergent, pharmaceutical, and medical (Prem et al., 2020). In industry, bacteria and fungi are the best biological sources of lipase (Boutaiba et al., 2006; Moura et al., 2015; Elif Demirkan et al., 2021). Compared to *Achromobacter*, *Alcaligenes*, *Arthrobacter*, *Burkholderia*, *Chromobacterium*, *Geobacillus*, and *Pseudomonas*, *Bacillus* plays a pivotal role in secreting lipase (Sarmah et al., 2018; Elif Demirkan et al., 2021). Since the leaves that Lepidopteran insects feed on contain fat or fatty acid, it is possible to screen for lipase-producing strains in their intestinal tracts. Nine lipase-producing bacterial strains, including *Bacillus, Brevibacterium, Corynebacterium, Staphylococcus, Klebsiella*, and *Stenotrophomonas* were obtained from the intestines of *Bombyx mori* (Wei et al., 2011). This is similar to the bacterial strains observed in the guts of *E. grisescens*. In addition, many lipase-producing strains were detected in the guts of *Antheraea assamensis, Helicoverpa armigera*, and *Plutella xylostella*, demonstrating their importance to host nutrition (Gandotra et al., 2016). However, the increased abundance of *Bacillus* and its functions under starvation stress have not previously been reported.

Previous studies have investigated insect behavior and their physiological responses to starvation stress (Ojima et al., 2018; Billings et al., 2018; Rovenko et al., 2015). With the development of sequencing technology and the rise of research in the field of gut microbes, researchers have found that intestinal microbes play an irreplaceable role in lipid metabolism. Previous studies conducted on germ free mice (GF-mice) and conventionally raised mice (CONV-R) found that intestinal bacteria help hosts to accumulate fat from food (Backhed et al., 2004). In-depth molecular research has shown that intestinal microbes can affect lipid metabolism by regulating adipose-related genes expression in mice (Zhao et al., 2018). Furthermore, a non-dominant intestinal bacterium of Niletilapia, *Citrobacter* spp., has been shown to aid the host in harvesting energy from a high-fat diet (Zhang et al., 2020). Here, we used deep sequencing technology and found that the significant change in EG-Q3 abundance in the gut of *E. grisescens* under starvation stress is related to lipid metabolism. This suggests that expanding the study of lipid metabolism in entomology may have benefits. This is the first study to explore the relationship between gut bacteria or non-core bacteria and insect lipid metabolism. In this study, 16S rRNA amplicon sequencing analysis showed that the abundance of *Bacillus* increased over time in the gut of starvation-stressed hosts and reached its maximum after 4 h of starvation. This implies that *Bacillus* participates in host lipid metabolism under starvation stress. In the *in vivo* test, group II significantly reduced body weight after 4 h of starvation relative to group I, likely due to the colonization of EG-Q3 in the gut. Therefore, EG-Q3 from *Bacillus* played an important role by helping *E. grisescens* consume its own fat bodies to obtain energy during the period of starvation. However, the difference in the rate of weight loss between the two groups decreased after 12 h. This may have been a result of proliferation of the small amount of *B. cereus* in normal intestines after starvation. This explanation is consistent with the changes in gut symbiotic bacterial communities found in our bioinformatics analysis.

In conclusion, the role of symbiotic bacteria should not be neglected in future studies of insect adaptation to starvation. Strengthening the research on the role of symbiotic bacteria and exploring intestinal bacteria related to stress resistance of other insects is conducive to a comprehensive and in-depth understanding of insect adaptation.

## Data Availability Statement

The datasets presented in this study can be found in online repositories. The names of the repository/repositories and accession number(s) can be found below: https://www.ncbi.nlm.nih.gov/genbank/, PRJNA720281.

## Author Contributions

YL, YY, and YoZ designed the project. XL, QS, TG, and LZ performed the experiments. YoZ and SL helped the analysis of sequencing data. XL and YoZ drafted the manuscript. YL, YuZ, and CW revised the manuscript. All authors contributed to the study conception and design and approved the final version for submission.

## Conflict of Interest

The authors declare that the research was conducted in the absence of any commercial or financial relationships that could be construed as a potential conflict of interest.

## Publisher’s Note

All claims expressed in this article are solely those of the authors and do not necessarily represent those of their affiliated organizations, or those of the publisher, the editors and the reviewers. Any product that may be evaluated in this article, or claim that may be made by its manufacturer, is not guaranteed or endorsed by the publisher.
