# Supplementary figures and images for: Bacillus cereus (EG-Q3) in the Gut of Ectropis grisescens Contributes to Host Response to Starvation Conditions
Source: Front Microbiol. 2022 Apr 11;13:785415. doi: 10.3389/fmicb.2022.785415 (PMC9037983; doi:10.3389/fmicb.2022.785415)

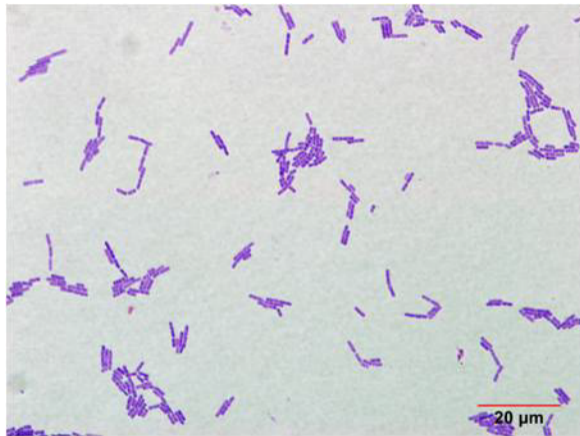

EG-Q3

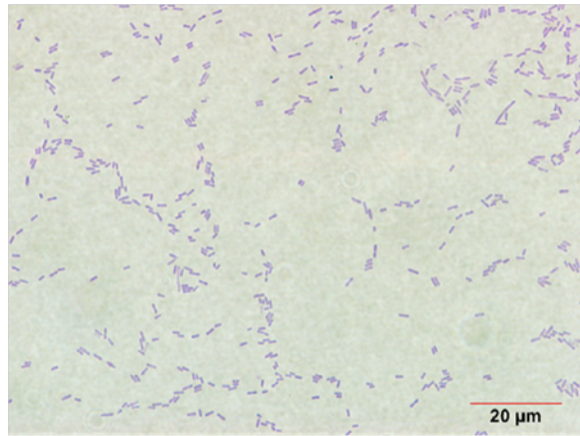

EG-Q4

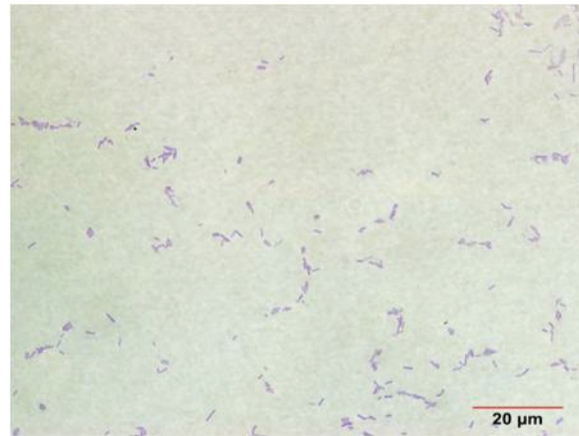

EG-Q5

Supplement: Supplementary file 1 [file Data_Sheet_1.PDF]
